# Improving engagement with preconsult patient-reported outcome measures: A mixed-methods pilot study

**DOI:** 10.1016/j.jdin.2026.08.016

**Published:** 2026-09-16

**Authors:** Chen He, Ying Shan Cheung, Jia Yi Chua, Qasrina Lim, Ellie Choi

**Affiliations:** a Division of Dermatology, Department of Medicine, National University Hospital, Singapore; b Department of Medicine, Yong Loo Lin School of Medicine, National University of Singapore, Singapore; c Centre for Research in Health Systems Performance, Yong Loo Lin School of Medicine, National University of Singapore, Singapore; d Centre for Behavioural and Implementation Science Interventions, Yong Loo Lin School of Medicine, National University of Singapore, Singapore, Singapore

**Keywords:** attitudes, health behavior, health knowledge, health outcomes, health services research, interview, motivation, patient reported outcomes, patient-centered care, practice, psychological/methods

*To the Editor:* Patient-reported outcome measures (PROMs) are tools completed by patients to directly report symptoms and quality of life, supporting patient-centered care.^1^^,^^2^ Their clinical value, however, is limited by patient noncompletion.^3^^,^^4^ This mixed-method study evaluated simple messaging-focused strategies to improve preconsult PROMs completion and explored broader implementational barriers in an outpatient dermatology setting.

Within each clinic list, dermatology outpatients were individually randomized to either an intervention arm testing a specific messaging strategy or a control arm using a standard template. PROM completion rates were compared between groups and supplemented by qualitative interviews and observations. This process was repeated across 6 separate comparisons evaluating different strategies, enabling independent pairwise estimation of each strategy’s effect while avoiding clinic-level clustering (Supplementary Methods, available via Mendeley at https://data.mendeley.com/datasets/cs389xczz8/3).

Interventions included (tested individually) as follows:

• Reminders 1 day before appointment if PROMs remained incomplete
• Visual aids explaining purpose of PROMs
• Personalized messages including patient and clinician names
• Alternative delivery channel: Short Message Service (compared to WhatsApp)
• Different message timings (eg, 1 day vs 3 days before appointment)
• A combined approach incorporating multiple features

The control strategy involved sending the study questionnaire weblink via WhatsApp 2 days before the appointment to the patient’s registered phone number.

Three hundred seventy-one patients were recruited between June and September 2025 (Table I). PROMs completion rates varied across strategies, with the highest in the reminder arm (57.1%) and lowest in the SMS arm (19.4%), while control rates ranged from 17.1% to 53.8% (Table I). Completion was higher among younger patients and lower among review patients (Multivariable logistic regression, Supplementary Table II, available via Mendeley at https://data.mendeley.com/datasets/cs389xczz8/3), possibly reflecting greater digital familiarity among younger patients, and greater caution toward an unexpected questionnaire among review patients.

**Table I:** PROMs completion rates by messaging strategy and corresponding control condition

Strategy	Intervention arm (*n* = 187 patients)∗			Control arm (*n* = 184 patients)†		
	Description	Patients, *n*	Completion rate (%)	Description	Patients, *n*	Completion rate (%)
Reminder	Reminder sent 1 d before appointment if PROMs remained incomplete	28	16/28 (57.1)	No reminder sent	26	14/26 (53.8)
Visual aids	Infographic with visual explanation of PROMs attached to message	26	10/26 (38.5)	No visual aids attached	26	9/26 (34.6)
Message delivery channel	Message delivered via Short Message Service (SMS)	31	6/31 (19.4)	Message delivered via Web-based tool (WhatsApp)	31	8/31 (25.8)
Message personalization	Personalized message including patient’s name, doctor’s name and appointment details	34	11/34 (32.4)	Generic message without personalization	35	6/35 (17.1)
Message timing	Message sent 1 d before appointment	28	10/28 (35.7)	Message sent 3 d before appointment	27	9/27 (33.3)
Combination of multiple strategies	Personalized WhatsApp message with visual aids sent 2 d before appointment, with reminder 1 d before if PROMs remained incomplete	40	17/40 (42.5)	Generic message sent 2 d before appointment with no reminders	39	13/39 (33.3)

*PROMs*, Patient-reported outcome measures.

∗ Six strategies were evaluated independently during the study period. For each strategy, patients on the clinic list during the corresponding study period were randomly assigned to either the intervention or control group, with the intervention differing only by the strategy implemented. A total of 371 patients were included in the study.

† For each individual-feature comparison, the intervention differed from its paired control only in the messaging feature evaluated. All control conditions consisted of a generic WhatsApp message containing a PROMs survey link sent 2 days prior to the scheduled appointment, without personalization, visual aids, or reminder prompts. The exception was messaging timing control, where the message was sent 3 days prior, instead of 2.

One hundred thirty-two patients were interviewed for qualitative feedback (Table II). Key facilitators included English proficiency, digital literacy, and the credibility of the messaging source. Barriers included irrelevance of questions, the perception that PROMs served research rather than clinical purposes, and workflow mismatches, such as caregivers receiving messages meant for patients and appointment lists not reflecting last-minute changes.

**Table II:** Key facilitators and barriers obtained from qualitative interviews with 132 patients∗

Theme	Representative observations or quotes	Implications
Language proficiency and digital literacy	Elderly Chinese-speaking participant shared that she “does not use WhatsApp” and “depends on her daughter to check messages”. Link did not render as clickable; required manual copy-paste.	1) Offer surveys in other languages. 2) Send the survey link as a separate message for easier copying.
Trustworthiness, credibility and data security	A participant perceived the message as a potential scam until reassured by the inclusion of the treating physician’s name and appointment details. “Normally (I) will not do any surveys via WhatsApp or SMS.” “Mobile number appeared random… sent on a Sunday (was) also slightly suspicious”	1) Incorporate personalized details such as clinician’s name or institution to improve credibility and patient trust 2) Notify in advance “face to face.” 3) Integrate PROMs into official hospital platforms or applications that have verification systems (eg, government-authenticated) to improve credibility.
Message delivery and design	“I don’t usually check SMS in general and would prefer WhatsApp or the official hospital app.” “Sending [reminders] 1 day before is good… captures the most ‘updated state’ of patient’s condition” “Infographics helped convey the purpose of questionnaires clearly.” “Was busy when link was sent; [I] only completed after reminder.”	1) Consider alternative communication platforms based on patient preferences 2) Schedule messages closer to clinic visits 3) Incorporate visual aids to improve patient engagement 4) Use reminder prompts to improve completion rates
PROMs relevance and usability	A patient reported that some questions felt less ‘relevant’ during an initial consultation, as her condition ‘doesn’t affect (her) emotionally’.	Including fields with stronger clinical relevance to derm patients, esp. for first visit patients (eg, optional photo uploads of affected skin).
Clinical workflow integration and operational processes	Some patients reported that messages were received by the next of kin rather than the patient (though this was appropriate for minors and elderly patients who rely on caregivers to complete PROMs).	Provide alternative access pathways, such as introducing QR codes in clinics to allow direct patient access and facilitate future survey access.
Patient motivation and perceived value	PROMs viewed as for research and not personally beneficial, “People usually don't feel that they'll benefit till they've seen a doctor”.	Develop a feedback loop to demonstrate how PROMs responses are incorporated into clinical care and decision-making.

*PROMs*, Patient-reported outcome measures; *SMS*, Short Message Service.

∗ Facilitators and barriers to PROMs completion were identified through onsite qualitative interviews with dermatology outpatients and analyzed using thematic analysis of interview data.

This study suggests that simple, low-resource strategies such as reminders and personalization may modestly enhance patient engagement in PROMs as 1 component of a broader implementation strategy. Additional strategies include providing feedback on how responses are used, leveraging social norms, and framing noncompletion as missed benefits (loss aversion).^5^ These strategies are particularly practical at the clinician/clinic level, complementing equally important system-level efforts to address structural barriers such as workflow, electronic medical record integration, digital access, and clinician engagement. Limitations include the small sample size in each strategy arm, which limited statistical testing but allowed a broader descriptive evaluation across more strategies.

## Conflicts of interest

None disclosed.
